# Point-of-Care Testing and Biomarkers in Biliary Diseases: Current Evidence and Future Directions

**DOI:** 10.3390/jcm14196724

**Published:** 2025-09-24

**Authors:** Jang Han Jung, Kyong Joo Lee, Se Woo Park, Dong Hee Koh, Jin Lee

**Affiliations:** Division of Gastroenterology, Department of Internal Medicine, Hallym University Dongtan Sacred Heart Hospital, Hallym University College of Medicine, Hwaseong 18450, Republic of Korea; con2000@hallym.or.kr (J.H.J.); britnepak@hallym.or.kr (S.W.P.); dhkoh@hallym.or.kr (D.H.K.); jinlee@hallym.or.kr (J.L.)

**Keywords:** biliary tract disease, point-of-care testing, diagnostic biomarkers, diagnosis, cholangiocarcinoma, molecular diagnostics, early diagnosis

## Abstract

Biliary tract diseases, including both benign and malignant conditions such as cholangitis, cholelithiasis, primary sclerosing cholangitis, cholangiocarcinoma, and gallbladder cancer, present significant challenges for timely diagnosis and effective clinical management. Conventional diagnostic approaches, which primarily rely on imaging and standard laboratory tests, often lack the sensitivity and specificity needed for early detection, accurate risk stratification, and personalized treatment planning. In recent years, advancements in point-of-care (POC) diagnostic technologies, along with the identification and validation of novel biomarkers, have begun to reshape the diagnostic landscape. This review provides a comprehensive overview of the clinical utility and limitations of current POC tests and biomarkers, ranging from well-established markers such as carbohydrate antigen 19-9 (CA19-9) and carcinoembryonic antigen (CEA) to emerging molecular indicators such as circulating microRNAs and circulating tumor DNA. We examine their applications across acute management, chronic disease monitoring, and cancer detection; identify existing gaps in diagnostic practice; and discuss strategies for incorporating these tools into standard clinical workflows to enhance patient outcomes.

## 1. Introduction

Biliary tract diseases comprise a diverse group of benign and malignant disorders affecting the biliary ductal system and gallbladder [1,2]. Benign conditions, such as acute and chronic cholangitis, cholelithiasis, and biliary strictures, are common and contribute significantly to global morbidity and hospital admissions [3,4]. In contrast, malignant diseases, including cholangiocarcinoma and gallbladder cancer, are less prevalent but are often diagnosed at advanced stages, leading to poor prognosis and a significant clinical burden [1].

Differentiating between benign and malignant biliary diseases remains challenging owing to overlapping clinical features, including jaundice, abdominal pain, and systemic inflammatory signs [5]. Early and accurate diagnosis is essential to guide appropriate treatment strategies and improve patient outcomes. However, conventional diagnostic tools, such as ultrasonography, computed tomography (CT), magnetic resonance cholangiopancreatography (MRCP), and standard laboratory tests, often demonstrate limited sensitivity and specificity. These limitations are particularly evident in early-stage disease or when distinguishing malignant from benign biliary strictures [6,7].

The growing emphasis on precision medicine has accelerated the development of novel diagnostic approaches that prioritize speed, accuracy, and personalized care. Point-of-care (POC) testing refers to diagnostic assays performed at or near the site of patient care and can deliver rapid, clinically actionable results, which are especially valuable in acute and outpatient settings [8,9,10]. Concurrently, advances in molecular diagnostics have led to the identification of a wide range of biomarkers, including protein markers such as carbohydrate antigen 19-9 (CA19-9) and carcinoembryonic antigen (CEA), nucleic acids such as circulating microRNAs (miRNAs) and cell-free DNA, and various metabolites detectable in serum, bile, or tissue [11,12]. These biomarkers offer promising potential for early detection, improved risk stratification, prognostication, and treatment response monitoring in both benign and malignant biliary diseases [13].

Moreover, artificial intelligence (AI) and machine learning algorithms are increasingly being integrated with imaging and biomarker data to enhance diagnostic performance. These technologies can improve pattern recognition, support risk prediction, and enable individualized clinical decision-making pathways based on multimodal data sources and are set to revolutionize the diagnostic landscape [14,15,16]. Despite these advancements, significant unmet needs remain, including the validation and standardization of emerging diagnostics, demonstration of clinical utility in diverse populations, and resolution of practical barriers to implementation in real-world settings.

This review presents a comprehensive overview of current and emerging roles of POC testing and biomarkers in the clinical management of biliary tract diseases. It examines their diagnostic and prognostic applications, highlights recent technological innovations including AI-assisted tools, and discusses key research priorities and implementation challenges in this rapidly evolving field.

## 2. Epidemiological and Clinical Overview of Biliary Tract Diseases

### 2.1. Epidemiology and Clinical Burden

Biliary tract diseases encompass a wide spectrum of hepatobiliary disorders that collectively contribute to significant global morbidity and healthcare resource utilization (Table 1) [17]. Gallstone disease is highly prevalent worldwide, affecting both Western and Asian populations, and frequently leads to complications such as acute cholecystitis and ascending cholangitis [18]. Although malignant biliary diseases, including cholangiocarcinoma and gallbladder carcinoma, occur less frequently, they are associated with markedly higher mortality rates, primarily due to their insidious onset, nonspecific clinical presentations, and frequent diagnosis at advanced stages [1,19].

Several risk factors predispose individuals to biliary tract malignancies. Chronic cholestasis, primary sclerosing cholangitis (PSC), choledochal cysts, hepatolithiasis, and prolonged biliary inflammation have all been consistently associated with increased cancer risk [20,21]. Notably, the clinical and radiological overlap between benign inflammatory conditions and malignant transformations often complicates diagnosis, posing a substantial challenge in clinical practice.

### 2.2. Diagnostic Challenges in Clinical Practice

The symptoms of biliary tract diseases are often nonspecific and overlap significantly between benign and malignant etiologies. Common presentations, such as abdominal pain, jaundice, pruritus, fever, and weight loss, lack adequate discriminatory value when considered individually. Although conventional imaging techniques, including transabdominal ultrasound, CT, and magnetic resonance cholangiopancreatography (MRCP), form the cornerstone of anatomical assessment, their capacity for functional or molecular characterization of lesions remains limited.

Similarly, standard laboratory tests measuring liver enzymes, bilirubin, and inflammatory markers often suffer from low specificity, particularly in patients with coexisting liver dysfunction or systemic inflammation [22,23]. Thus, these diagnostic tools frequently fail to distinguish malignant strictures or tumors from benign inflammatory or obstructive processes, underscoring the need for improved diagnostic accuracy.

### 2.3. Unmet Needs in Biliary Diagnostics

Given the limitations of current diagnostic workflows, there is an urgent need for tools that deliver rapid, reliable, and noninvasive diagnostic information at the point of care. Ideally, such approaches would enable early detection of biliary tract diseases, support accurate risk stratification, and facilitate timely therapeutic decision making.

Point-of-care testing and molecular biomarker profiling have emerged as promising adjuncts to conventional imaging and laboratory evaluations. These methods can offer real-time diagnostic insights, potentially improving clinical outcomes by reducing diagnostic delays and enabling personalized patient management.

## 3. Point-of-Care Testing in Biliary Tract Disease

### 3.1. Principles and Clinical Utility

POC testing refers to diagnostic assays conducted at or near the site of patient care that can deliver results within minutes. Such testing facilitates immediate clinical decision making by circumventing delays associated with centralized laboratories. In the context of biliary tract disease, this is particularly valuable during acute presentations, such as suspected cholangitis or biliary sepsis, where timely diagnosis and intervention can significantly reduce morbidity and mortality [24].

**Figure 1:**
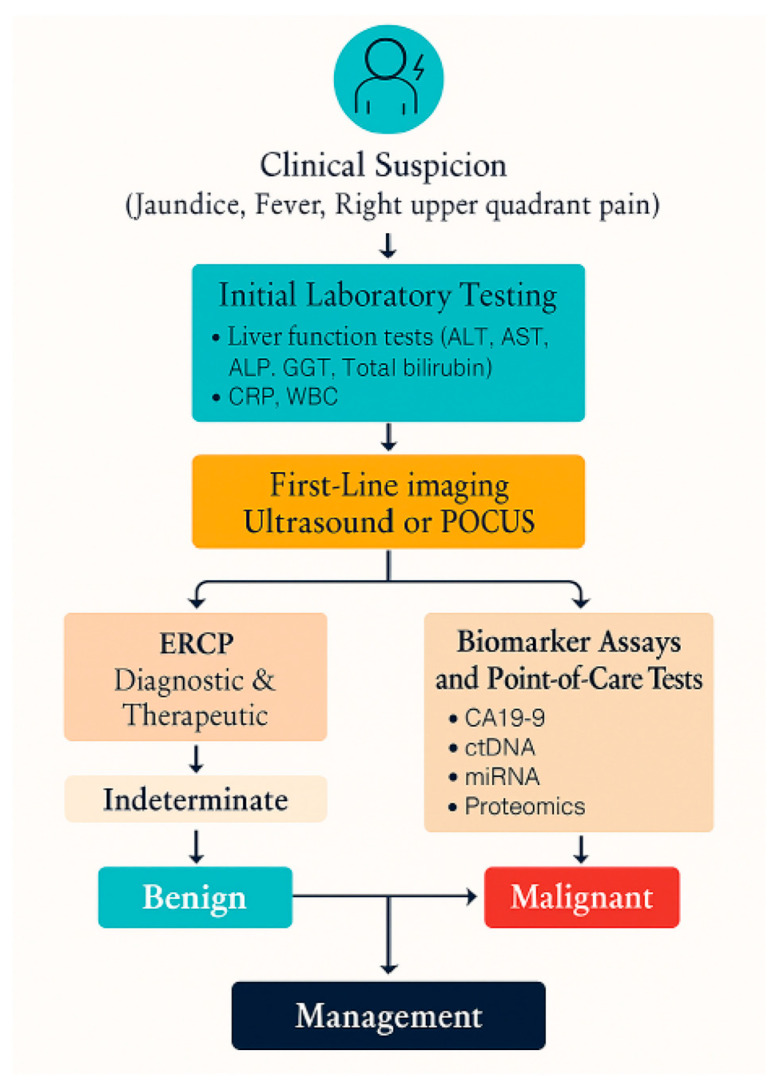
Diagnostic algorithm for suspected biliary disease. This figure illustrates a proposed diagnostic algorithm for evaluating patients with suspected biliary disease, highlighting the potential integration of point-of-care tests and biomarkers into conventional imaging and laboratory workflows. ALP, alkaline phosphatase; ALT, alanine aminotransferase; AST, aspartate aminotransferase; CA19-9, carbohydrate antigen 19-9; CRP, C-reactive protein; ctDNA, circulating tumor DNA; ERCP, endoscopic retrograde cholangiopancreatography; GGT, gamma-glutamyl transferase; miRNA, microRNA; POCUS, point-of-care ultrasound; and WBC, white blood cell.

A spectrum of POC technologies, ranging from bedside biochemical panels and inflammatory markers to advanced molecular diagnostics and real-time on-site cytological assessments, is either commercially available or under active clinical investigation for biliary tract disease [12,25]. An integrated diagnostic algorithm summarizing their clinical applications is illustrated in Figure 1. Table 2 summarizes the available and emerging POC modalities, their clinical utility, turnaround times, and limitations.

### 3.2. Current Point-of-Care Applications

#### 3.2.1. Liver Enzyme and Function Panels

**Figure 2:**
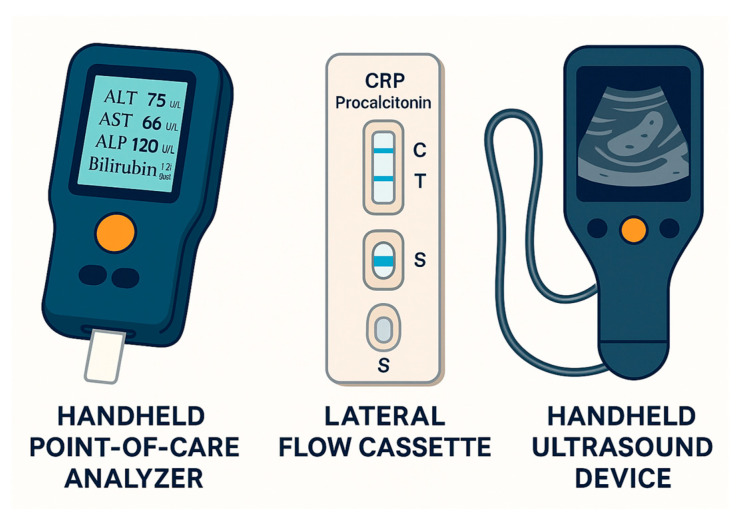
Handheld point-of-care testing devices commonly used in biliary diagnostics. (**Left**) liver enzyme analyzers measuring ALT, AST, ALP, and bilirubin; (**Middle**) lateral flow immunoassays for inflammatory markers such as CRP and procalcitonin; (**Right**) portable handheld ultrasound devices for rapid biliary tract imaging. ALT, alanine aminotransferase; AST, aspartate aminotransferase; ALP, alkaline phosphatase; and CRP, C-reactive protein.

Handheld or bedside analyzers capable of measuring liver enzymes such as alanine aminotransferase (ALT), aspartate aminotransferase (AST), alkaline phosphatase (ALP), gamma-glutamyl transferase (GGT), bilirubin, and albumin are now commercially available and widely used in emergency departments and hospital wards (Figure 2). These tools enable rapid triage and risk stratification of patients presenting with jaundice, abdominal pain, or suspected biliary obstruction. For example, in an emergency setting, elevated direct bilirubin and ALP levels from a bedside POC analyzer may prompt urgent imaging or consultation for potential ERCP [26].

#### 3.2.2. Inflammatory and Infectious Markers

POC assays for markers such as C-reactive protein (CRP) and procalcitonin are increasingly utilized for the diagnosis and monitoring of bacterial cholangitis and the assessment of infection severity [27]. Some modern systems include automated leukocyte (white blood cell, WBC) and differential counts [28]. For instance, a combination of elevated CRP levels and WBC counts may reinforce the suspicion of acute ascending cholangitis and justify the prompt initiation of antibiotics and biliary decompression.

#### 3.2.3. Bedside Ultrasound

POC ultrasonography (POCUS) has become a cornerstone for the rapid assessment of patients with suspected biliary diseases [29]. Internists and gastroenterologists now frequently use handheld ultrasound devices to detect gallstones, biliary duct dilation, and gallbladder wall thickening and to assess complications such as abscesses or pericholecystic fluid. When incorporated into early clinical workflows, POCUS facilitates expedited diagnosis and intervention, particularly in unstable or critically ill patients. However, its sensitivity is operator-dependent and may be reduced in patients with obesity or excess bowel gas. Although POC tools and novel biomarkers are increasingly incorporated into practice, major international guidelines continue to differ in their diagnostic recommendations for biliary tract diseases [30].

Table 3 provides a comparative summary of key guidelines from the American Association for the Study of Liver Diseases (AASLD), European Association for the Study of the Liver (EASL), Asian Pacific Association for the Study of the Liver (APASL), and the Tokyo Guidelines 2018, and it highlights opportunities for integrating POC testing and biomarker strategies into established diagnostic frameworks [4,31,32,33]. Across current international guidelines, diagnostic pathways remain focused on conventional clinical, biochemical, and imaging approaches. While AASLD and EASL mention CA19-9 (with or without CEA) as supportive markers in suspected cholangiocarcinoma, they stop short of recommending novel biomarkers. APASL guidelines emphasize early ERCP in obstructive cholangitis, reflecting regional disease patterns, but provide little guidance on biomarker use. The Tokyo Guidelines 2018 prioritize clinical and laboratory criteria for acute cholangitis severity assessment, without incorporating biomarkers.

Overall, none of the guidelines formally recommend POC testing or novel biomarkers, highlighting a consistent gap between technological advances and clinical guideline integration. This underscores the urgent need for multicenter validation and consensus to incorporate POC testing and biomarker strategies into standardized biliary disease management.

#### 3.2.4. Rapid On-Site Evaluation (ROSE)

Endoscopic ultrasound-guided fine-needle aspiration (EUS-FNA) is an established method for sampling masses and strictures of the bile duct and adjacent structures [34,35]. The integration of ROSE, in which a cytopathologist or trained technologist assesses specimen adequacy and provides a preliminary diagnosis during the procedure, enables real-time feedback to the endoscopist [36]. This approach facilitates adjustments in the target site, needle type, or number of passes, increasing the diagnostic yield and often obviating the need for repeat procedures [37].

Multiple studies have shown that ROSE during EUS-FNA improves diagnostic accuracy, shortens the time to a definitive diagnosis, and reduces unnecessary additional procedures in patients with indeterminate biliary strictures or suspected malignancy [38,39,40]. In settings without on-site cytopathology, alternatives, such as telecytology and rapid staining kits, are under investigation [41]. Limitations include the need for specialized personnel and workflow implications. Nevertheless, ROSE is increasingly reflected in clinical practice guidelines for optimizing tissue acquisition in biliary tract and pancreatic neoplasms.

### 3.3. Emerging Molecular POC Technologies

The frontier of POC diagnostics has advanced rapidly with the advent of microfluidic devices, biosensors, and nucleic acid amplification technologies [42,43]. These platforms can detect bacterial DNA, viral RNA, and cancer-specific somatic mutations in minuscule volumes of blood, bile, or urine, potentially enabling pathogen identification or early cancer detection within an hour [44].

While still under investigation, several pilot studies demonstrate their utility—such as PCR-based identification of causative bacteria in septic cholangitis [45], or lab-on-a-chip mutation panels for rapid detection of common cholangiocarcinoma mutations (Kirsten rat sarcoma viral oncogene homolog (KRAS), isocitrate dehydrogenase 1 and 2 (IDH 1/2), fibroblast growth factor receptor 2 (FGF2), tumor protein p53 (TP53)) [46,47]. Portable miRNA chips also show promise for distinguishing malignant from benign strictures but require further clinical validation and standardization before widespread adoption.

### 3.4. Limitations and Practical Considerations

While POC tests offer clear advantages in biliary disease management, including rapid results and support for timely decision making, several limitations must be considered. First, the accuracy of many POC assays may be lower than that of central laboratory methods, with variable sensitivities and specificities [48]. This limitation can lead to occasional false-positive or false-negative results, making clinical interpretation and confirmatory testing essential.

Operational challenges also persist. POC devices require regular calibration and staff training to ensure reliability. Lack of experience or poor technique may affect validity, particularly for operator-dependent tools such as POC ultrasound [49]. The cost of purchasing, maintaining, and supplying consumables for POC equipment can be a significant barrier, especially in resource-limited environments where funding and reimbursement are uncertain.

In addition, workflow integration remains challenging. Incorporating POC results into existing clinical protocols and electronic health records is not always seamless, and additional steps may complicate busy clinical settings [50]. Furthermore, although novel POC technologies—such as molecular and genetic assays—show promise, their widespread adoption requires further validation in larger studies.

Ultimately, POC tests should complement rather than replace comprehensive diagnostic assessments. Clinicians must interpret results in context and confirm findings with standard laboratory or imaging studies when appropriate.

## 4. Biomarkers in Biliary Tract Disease

### 4.1. Clinical Significance and Role of Biomarkers

Biomarkers are quantifiable indicators of biological processes, pathological states, or response to therapy that are measurable in blood, bile, tissue, or other body fluids. In biliary tract diseases, they are increasingly central to early diagnosis, risk stratification, prognostication, and monitoring of treatment response. Effective biomarkers help differentiate benign from malignant processes, anticipate disease progression, and detect recurrence earlier than imaging or clinical symptoms. Their use is expanding alongside precision-medicine strategies (Table 4).

### 4.2. Established Serum Biomarkers

#### 4.2.1. Carbohydrate Antigen (CA19-9)

CA19-9 is the most widely used serum biomarker for cholangiocarcinoma and pancreatic cancer [51,52]. Its clinical utility is limited by suboptimal specificity, as levels may be elevated in obstructive jaundice, cholangitis, or pancreatitis, and by false negatives in Lewis antigen-negative individuals (approximately 5–10%), who do not synthesize the antigen [53].

#### 4.2.2. Carcinoembryonic Antigen (CEA)

CEA is sometimes measured in combination with CA19-9, but its low sensitivity and specificity, and its elevation in multiple benign and malignant conditions, restrict its value as a standalone test in biliary tract cancers [53].

#### 4.2.3. Cholestatic Enzymes (ALP and GGT)

Alkaline phosphatase and GGT are classic indicators of cholestasis; however, they cannot reliably distinguish between malignant and benign biliary diseases [54].

### 4.3. Novel and Emerging Biomarkers

#### 4.3.1. Circulating- and Bile-Derived miRNAs

Circulating and bile-derived miRNAs, notably miR-21, miR-221, and members of the miR-200 family, are stable in body fluids and show disease-specific upregulation in cholangiocarcinoma [55]. When combined into multianalyte panels, these markers can support early, noninvasive discrimination between malignant and benign biliary strictures [55]. Among emerging biomarkers, for example, a large single study combining plasma miRNAs (miR-21, miR-122, and CA19-9) achieved a sensitivity of 73.0% and specificity of 87.4% (AUC 0.853) for distinguishing intrahepatic cholangiocarcinoma from non-malignant controls [56]. Similarly, bile miRNA signatures in the MIRABILE study produced an AUC of 0.79 (95% CI 0.70–0.88), sensitivity of 65%, and specificity of 82% in differentiating malignant versus benign pancreaticobiliary disease [57]. Despite these encouraging findings, significant heterogeneity among study cohorts, variability in biospecimen handling, and lack of standardized cutoff values continue to limit the generalizability of results. Most available data are derived from retrospective or single-center studies, and large, multicenter prospective validations are still lacking.

Importantly, current international guidelines do not yet incorporate miRNA-based assays into diagnostic pathways, underscoring the need for rigorous validation before clinical implementation. Nevertheless, these markers represent promising candidates for future integration into guideline-based biliary disease management once reproducibility and real-world applicability are confirmed.

#### 4.3.2. Bile-Derived Protein Biomarkers

Proteins such as MUC5AC, CEACAM6, MUC4, and NGAL, quantified from bile (often collected during ERCP), demonstrate promising diagnostic accuracy [58,59]. However, routine use is constrained by the invasiveness, cost, and logistics of bile sampling.

#### 4.3.3. Liquid Biopsy: ctDNA in Plasma and Bile

Next-generation sequencing enables detection of tumor-derived alterations, such as KRAS, IDH1/2, FGFR2, and TP53, in plasma or bile (“liquid biopsy”) [11,60]. This approach is promising for early detection, molecular profiling, and selection of targeted therapies. For example, in a study by Rizzo et al., a ctDNA panel demonstrated a specificity of approximately 93%, though sensitivity was variable and generally lower in early or low-tumor-burden cases [61]. Other reports have similarly indicated that sensitivity often ranges between 50–70% for key driver mutations in plasma samples, reflecting the challenges of tumor heterogeneity and low mutant allele frequency in early disease [62]. Importantly, most existing studies are limited by small sample sizes and retrospective design, and multicenter prospective trials are required to confirm real-world clinical utility. While validation is ongoing, liquid biopsy approaches may supplement or, in select scenarios, substitute for tissue-based diagnostics.

#### 4.3.4. Emerging Serum Protein Biomarkers

Emerging protein candidates, including leucine-rich α-2-glycoprotein (LRG), osteopontin, and pyruvate kinase M2 (PKM2), show diagnostic and prognostic potential. Robust multicenter validation is needed before clinical adoption [59,63].

### 4.4. Multimodal and Imaging-Based Biomarker Strategies

#### 4.4.1. Combined Biomarker Panels and Risk Models

Integrative models that combine traditional serum proteins, miRNA signatures, tumor genetic alterations, and imaging features outperform single-marker approaches and are likely to become standard elements of future biliary diagnostic pathways.

#### 4.4.2. AI-Assisted Metabolic Imaging

Artificial intelligence-assisted, label-free 3D optical diffraction tomography can profile lipid-droplet features in biliary tract cells [64]. Machine learning models, such as EfficientNet, applied to 3D refractive index tomograms have demonstrated excellent diagnostic performance, with reported AUC values exceeding 0.99 in distinguishing cancerous from normal cholangiocytes in experimental studies [64]. Beyond simple classification, visualization techniques (e.g., LayerCAM) consistently highlight lipid droplet-rich regions that correspond to known cancer-associated metabolic phenotypes. Although these findings are highly promising, current evidence remains limited to preclinical and small-scale translational studies. Larger prospective trials and real-world validation will be essential to determine whether AI-assisted ODT can reliably complement cytology or liquid biopsy for rapid, label-free malignancy detection and personalized risk stratification.

### 4.5. Limitations and Future Directions

Despite substantial progress, routine clinical adoption of biliary biomarkers remains constrained by heterogeneous assays and the absence of harmonized decision thresholds, which undermine reproducibility and cross-study comparability. Many candidates still lack large, prospective, multicenter validation in demographically and etiologically diverse cohorts with prespecified clinical endpoints. Utility is further limited by reliance on invasive sampling (e.g., ERCP-derived bile), variable costs and reimbursement, and the operational challenges of embedding results into clinical workflows and electronic health records.

Future priorities are assay standardization and external quality assurance; development and validation of noninvasive or minimally invasive sampling strategies; and pragmatic trials that demonstrate improvements in time-to-treatment, procedure yield, and patient-centered outcomes. Integrating biomarkers with clinical variables, imaging features, and AI-enabled analytics may enhance discrimination and calibration and support personalized management. Equitable access, through cost-conscious design, interoperable implementation, and tiered testing pathways, will be essential to realize these benefits at scale.

#### 4.5.1. Clinical Implementation and Cost-Effectiveness

While novel POC assays and biomarker panels demonstrate strong diagnostic performance, their clinical value must be established through improvements in patient-centered outcomes and healthcare efficiency. Real-world studies indicate that rapid POC inflammatory marker testing in suspected acute cholangitis can shorten time to first antibiotics and to definitive biliary drainage, reducing length of stay and associated costs [26,65]. Similarly, embedding serum- or bile-derived biomarker panels into pre-procedural triage algorithms may lower the rate of non-therapeutic or avoidable invasive procedures [58,59]. However, broad adoption will require deliberate implementation: clinician education, standardized operating procedures, and rigorous quality-assurance/quality-control programs to ensure reproducibility across diverse healthcare settings.

#### 4.5.2. Integrating AI with Multi-Omics

Beyond single-assay tests, the next wave of biliary diagnostics will couple integrated multi-omics, namely proteomics, transcriptomics, metabolomics, lipidomics, and circulating tumor nucleic acids, with AI-enabled analytic pipelines. These models can assimilate serial patient data to continuously recalibrate individualized risk, informing surveillance intervals and therapeutic selection. Their clinical adoption will require rigorous, multicenter validation that quantifies incremental benefit over current pathways and establishes cost-effectiveness and operational feasibility across both high- and low-resource settings.

## 5. Future Directions

### 5.1. Technological Innovation

Recent advances in microfluidics, biosensors, and lab-on-a-chip platforms are enabling rapid, multiplexed diagnostics deployable at the bedside and, increasingly, in the home. Artificial intelligence, including machine- and deep-learning methods, is being applied to complex biomarker and imaging data. These integrated approaches have the potential to surpass traditional diagnostics by improving accuracy, enabling real-time risk stratification, and supporting point-of-care clinical decision making.

### 5.2. Multimarker and Integrated Models

Diagnostic strategies are likely to move beyond single analytes toward multimodal algorithms that integrate POC test results, molecular signatures, routine laboratory analytes, and imaging features. Such integrated models aim to enhance diagnostic precision, personalize treatment selection, and optimize monitoring and surveillance protocols for both benign and malignant biliary tract diseases.

### 5.3. Expanding Access and Equity

Achieving equitable access remains a major challenge, particularly in rural and resource-limited settings where advanced imaging and endoscopy are scarce. Simple, portable, and cost-effective diagnostic platforms with reliable performance are critical to reducing disparities worldwide. Strategically deployed innovations can improve early detection and longitudinal management of biliary diseases in underserved populations.

### 5.4. Research and Validation Priorities

To define the clinical impact of POC tests and biomarker-based diagnostics in biliary tract disease, future research should address several priorities. First, large, prospective, multicenter clinical trials are needed to evaluate whether biomarker- or POC-guided strategies improve key outcomes such as morbidity, mortality, time to intervention, and procedure yield. Second, systematic reviews, meta-analyses, and harmonization efforts should establish optimal biomarker panels, assay methodologies, and clinically actionable thresholds to facilitate standardization and comparability across institutions. Third, clear regulatory and reimbursement pathways are required to support safe, sustainable implementation through robust quality assurance and post-market performance monitoring. Finally, incorporating patient-reported outcomes and quality-of-life measures into clinical evaluations will clarify the broader impact of early diagnosis and minimally invasive monitoring. Coordinated efforts across basic, translational, and clinical research, aligned with supportive health-policy initiatives, will be essential to translate promising biomarkers and POC technologies into routine practice and improve outcomes in biliary tract disease.

## 6. Conclusions

Biliary tract diseases remain diagnostically challenging owing to their heterogeneous clinical presentations and the difficulty of distinguishing benign from malignant conditions. Early and accurate diagnosis is especially critical for malignancies such as cholangiocarcinoma, where timely intervention can significantly influence outcomes.

Point-of-care testing and novel molecular biomarkers are reshaping diagnostic and management pathways. While established serum markers, such as CA19-9 and CEA, retain value, emerging tools, including circulating miRNAs, bile-derived proteomic signatures, ctDNA, and AI-assisted metabolic or label-free imaging, offer enhanced diagnostic precision and clinically relevant prognostic information. When integrated with clinical assessment and imaging, these multimodal approaches can improve accuracy, enable near-real-time risk stratification, and inform personalized therapeutic strategies (see Table 4).

Despite rapid progress, several barriers must be addressed before these innovations can be adopted into routine practice: assay harmonization with clinically actionable, context-specific thresholds; prospective, multicenter validation using external (geographically distinct) cohorts; rigorous cost-effectiveness evaluation; and seamless integration into clinical workflows. Meeting these requirements will require coordinated investment in method standardization, laboratory and digital infrastructure, robust quality assurance, and clinician training.

Ultimately, integrating POC testing and advanced biomarker technologies into routine clinical practice has the potential to transform precision medicine for biliary tract diseases. When implemented within guideline-concordant, interoperable workflows, these tools can enable earlier diagnosis, support tailored treatment selection and improve long-term outcomes for patients with both benign and malignant biliary disorders.

The adoption of POC diagnostics and biomarker-based approaches into routine biliary tract disease management has the potential to improve diagnostic accuracy, shorten time to treatment, and enhance patient outcomes. Looking ahead, harmonized, society-endorsed guidelines that incorporate POC assays, near-patient imaging, and molecular biomarkers into standardized, interoperable workflows will be essential. Large, prospective, multicenter studies, complemented by real-world evidence, are needed to validate clinical utility across diverse care settings. Ultimately, the convergence of rapid diagnostics, digital health infrastructure, and precision-medicine strategies is expected to redefine the clinical landscape of biliary tract diseases by enabling earlier detection, individualized treatment, and more efficient resource utilization.

## Figures and Tables

**Table 1:** Overview of major biliary tract diseases and their key features.

Disease	Classification	Common Symptoms	Risk Factors	Prevalence	Prognosis
Acute cholangitis	Benign	Fever, jaundice, abdominal pain	Gallstones, biliary strictures	Common	Good with prompt treatment
Primary sclerosing cholangitis (PSC)	Benign	Jaundice, pruritus	Autoimmune conditions	Rare	Increased risk of malignancy
Gallstone disease	Benign	Abdominal pain	Obesity, advanced age	Very common	Excellent after cholecystectomy
Cholangiocarcinoma	Malignant	Jaundice, weight loss	PSC, choledochal cysts	Uncommon	Poor prognosis
Gallbladder cancer	Malignant	Abdominal pain, palpable mass	Gallbladder polyps	Rare	Poor prognosis

**Table 2:** Summary of point-of-care tests in biliary disease.

Test Type	Target Marker	Sample Type	Clinical Utility	Time to Result	Limitations
POC Liver Panel Analyzer	ALT, AST, ALP, GGT, Bilirubin, Albumin	Whole blood/Serum	Initial triage for biliary obstruction or hepatopathy	~10–15 min	May have lower accuracy than laboratory assays
POC Inflammatory Marker	CRP, Procalcitonin	Whole blood	Diagnosis and monitoring of cholangitis	~15–20 min	False positives in non-infectious inflammation
POC Leukocyte Panel	WBC count and differential	Whole blood	Sepsis screening in acute cholangitis	~10 min	Limited differential accuracy
POCUS (bedside ultrasound)	Gallstones, bile duct dilation, sludge	N/A (imaging tool)	Rapid bedside imaging for biliary tract assessment	Immediate	Operator-dependent; limited in obese patients
Microfluidic NAAT System	Bacterial DNA, Viral RNA	Bile/Blood/Urine	Pathogen identification in biliary infection	30–60 min	Not yet validated for routine biliary disease diagnostics
ctDNA Panel (lab-on-chip)	KRAS, IDH1/2, FGFR2, TP53 mutations	Plasma/Bile	Detection of biliary tract cancers	1–2 h	Costly, technically complex; not widely available
Biosensor-based miRNA Chip	miR-21, miR-221, miR-200 family	Serum/Bile	Early cancer detection, risk stratification	30–60 min	Under investigation; standardization and cutoff values lacking

ALP, alkaline phosphatase; ALT, alanine aminotransferase; AST, aspartate aminotransferase; CRP, C-reactive protein; ctDNA, circulating tumor DNA; FGFR2, fibroblast growth factor receptor 2; GGT, gamma-glutamyl transferase; IDH1/2, isocitrate dehydrogenase 1 and 2; KRAS, Kirsten rat sarcoma viral oncogene homolog; miRNA, microRNA; N/A, not applicable; NAAT, nucleic acid amplification test; POC, point-of-care; POCUS, point-of-care ultrasound; TP53, tumor protein p53; and WBC, white blood cell count.

**Table 3:** Comparison of international guidelines for biliary tract disease diagnosis and potential integration of POC tests and biomarkers.

Guideline	Primary Diagnostic Approach	Use of POC Tests	Use of Biomarkers	Specific Recommendations	Potential Role for Integration
AASLD (American Association for the Study of Liver Diseases) [31]	Clinical evaluation, LFTs, ultrasound as first-line; MRCP or EUS for indeterminate cases; ERCP for therapeutic purposes	Not routinely addressed	CA19-9 considered in suspected cholangiocarcinoma (esp. with PSC)	Emphasis on imaging + CA19-9 in select contexts	Incorporation of POC CRP/LFTs in acute cholangitis for rapid triage; biomarker panels for indeterminate strictures
EASL (European Association for the Study of the Liver) [4]	Stepwise approach: history/physical, LFTs, ultrasound → advanced imaging (MRCP, CT) → ERCP for diagnosis/therapy	Not specifically addressed	CA19-9, CEA mentioned; emphasize exclusion of benign causes	Support CA19-9 as adjunct but not standalone	Use of POC inflammatory markers to guide early management; miRNA or ctDNA to improve malignant vs. benign differentiation
APASL (Asian Pacific Association for the Study of the Liver) [33]	Emphasis on regional epidemiology; ultrasound as first-line; early ERCP for obstructive cholangitis; MRCP/EUS for non-urgent cases	Not specifically addressed	Limited mention; CA19-9 for cholangiocarcinoma suspicion	Prioritizes ERCP in high-incidence regions	POC LFTs/CRP in rural settings; bile-based proteomics for high-risk groups (e.g., hepatolithiasis, choledochal cysts)
Tokyo Guidelines 2018 (TG18; for Acute Cholangitis/Cholecystitis) [32]	Diagnostic criteria: systemic inflammation, cholestasis, imaging evidence	Indirectly: early labs and imaging encouraged	No formal biomarker recommendations	Structured severity grading with labs and imaging	Integration of POC CRP/WBC count for faster TG18 severity grading; emerging biomarkers for early sepsis risk prediction

CA19-9, carbohydrate antigen 19-9; CEA, carcinoembryonic antigen; CRP, C-reactive protein; CT, Computed tomography; ctDNA, circulating tumor DNA; ERCP, endoscopic retrograde cholangiopancreatography; EUS, endoscopic ultrasound; LFTs, liver function tests; miRNA, microRNA; MRCP, magnetic resonance cholangiopancreatography; POC, point-of-care; PSC, primary sclerosing cholangitis; and WBC, white blood cell.

**Table 4:** Summary of traditional and emerging biomarkers in biliary tract disease.

Biomarker	Sample Type	Disease Target	Limitations	Clinical Utility
CA19-9	Serum	CCC	False positive: cholestasis, Lewis-neg	Diagnosis, monitoring
CEA	Serum	CCC	Low specificity	Adjunctive, monitoring
miR-21, -221	Serum/bile	CCC, GB cancer	Under investigation, no standard cutoff	Early diagnosis
MUC5AC	Bile	CCC	Requires ERCP	Malignant vs. benign differentiation
ctDNA	Plasma/bile	CCC	Cost, technical complexity	Targeted therapy, prognosis

CA19-9, carbohydrate antigen 19-9; CCC, cholangiocarcinoma; CEA, carcinoembryonic antigen; GB cancer, gallbladder cancer.

## Data Availability

No new data were created or analyzed in this study. Data sharing is not applicable to this article.

## Abbreviations

The following abbreviations are used in this manuscript:

AASLD	American Association for the Study of Liver Diseases
ALP	Alkaline phosphatase
ALT	Alanine aminotransferase
APASL	Asian Pacific Association for the Study of the Liver
AST	Aspartate aminotransferase
AUC	Area under the curve
CA19-9	Carbohydrate antigen 19-9
CEA	Carcinoembryonic antigen
CEACAM6	Carcinoembryonic antigen-related cell adhesion molecule 6
CRP	C-reactive protein
CT	Computed tomography
ctDNA	Circulating tumor DNA
EASL	European Association for the Study of the Liver
ERCP	Endoscopic retrograde cholangiopancreatography
EUS	Endoscopic ultrasound
EUS-FNA	Endoscopic ultrasound-guided fine-needle aspiration
FGFR2	Fibroblast growth factor receptor 2
GGT	Gamma-glutamyl transferase
IDH1/2	Isocitrate dehydrogenase 1/2
KRAS	Kirsten rat sarcoma viral oncogene homolog
LFTs	Liver function tests
LRG	Leucine-rich alpha-2 glycoprotein
miRNA	Microrna
MRCP	Magnetic resonance cholangiopancreatography
MUC4	Mucin 4
MUC5AC	Mucin 5AC
NAAT	Nucleic acid amplification test
NGAL	Neutrophil gelatinase-associated lipocalin
PKM2	Pyruvate kinase M2
POC	Point-of-care
POCUS	Point-of-care ultrasound
PSC	Primary sclerosing cholangitis
ROSE	Rapid on-site evaluation
TG18	Tokyo guidelines 2018
TP53	Tumor protein p53
WBC	White blood cell count
